# Efflux modulation as an antimicrobial strategy: mechanisms and translational perspectives

**DOI:** 10.1038/s44386-026-00083-0

**Published:** 2026-10-12

**Authors:** Waleska Stephanie da Cruz Nizer, Cesar Augusto Roque-Borda, Chongbing Liao, Heitor Leocádio de Souza Rodrigues, Subham Kumar Vishwakarma, Zidane Qriouet, Andreia Bagliotti Meneguin, Fernando Rogério Pavan

**Affiliations:** 1 Tuberculosis research laboratory, São Paulo State University (UNESP), School of Pharmaceutical Sciences, Araraquara, Brazil; 2 Vicerrectorado de Investigación, Universidad Católica de Santa María, Arequipa, Peru; 3 Key Laboratory of Medical Molecular Virology (MOE/NHC/CAMS), Shanghai Institute of Infectious Disease and Biosecurity, School of Basic Medical Sciences, Fudan University, Shanghai, China; 4 State Key Laboratory of Molecular Engineering of Polymers, Department of Macromolecular Science, Fudan University, Shanghai, China; 5 São Paulo State University (UNESP), School of Agricultural and Veterinary Sciences, Department of Pathology, Reproduction and One Health (DPRSU), Jaboticabal, Brazil; 6 Department of Drugs and Medicines, São Paulo State University “Júlio de Mesquita Filho”, Araraquara, São Paulo Brazil

**Keywords:** Computational biology and bioinformatics, Microbiology

## Abstract

Efflux pumps are widely viewed as antibiotic resistance determinants, yet their roles in stress adaptation, virulence, quorum sensing, and biofilm formation make them context-dependent therapeutic targets. This Perspective distinguishes four efflux intervention strategies: direct inhibition, bioenergetic or membrane-mediated suppression, regulatory modulation, and dual-effect approaches. We examine why promising in vitro findings rarely translate clinically and argue that efflux-targeting strategies require defined therapeutic objectives and integrated multidisciplinary approaches.

## Introduction

Efflux pumps are versatile membrane transporters of bacterial cells that export a wide range of structurally diverse molecules and represent a major driver of antimicrobial resistance^1^. Although antimicrobial resistance and treatment failure are often multifactorial, increased efflux activity is commonly observed among resistant ESKAPEE (***E****nterococcus faecium*, ***S****taphylococcus aureus*, ***K****lebsiella pneumoniae*, ***A****cinetobacter baumannii*, ***P****seudomonas aeruginosa*, ***E****nterobacter* spp., and ***E****scherichia coli*) clinical isolates, where broad substrate specificity^2–4^, functional redundancy^5^, and dynamic expression^6,7^ enhance bacterial adaptability and limit the efficacy of antimicrobial therapies.

**Fig. 1:**
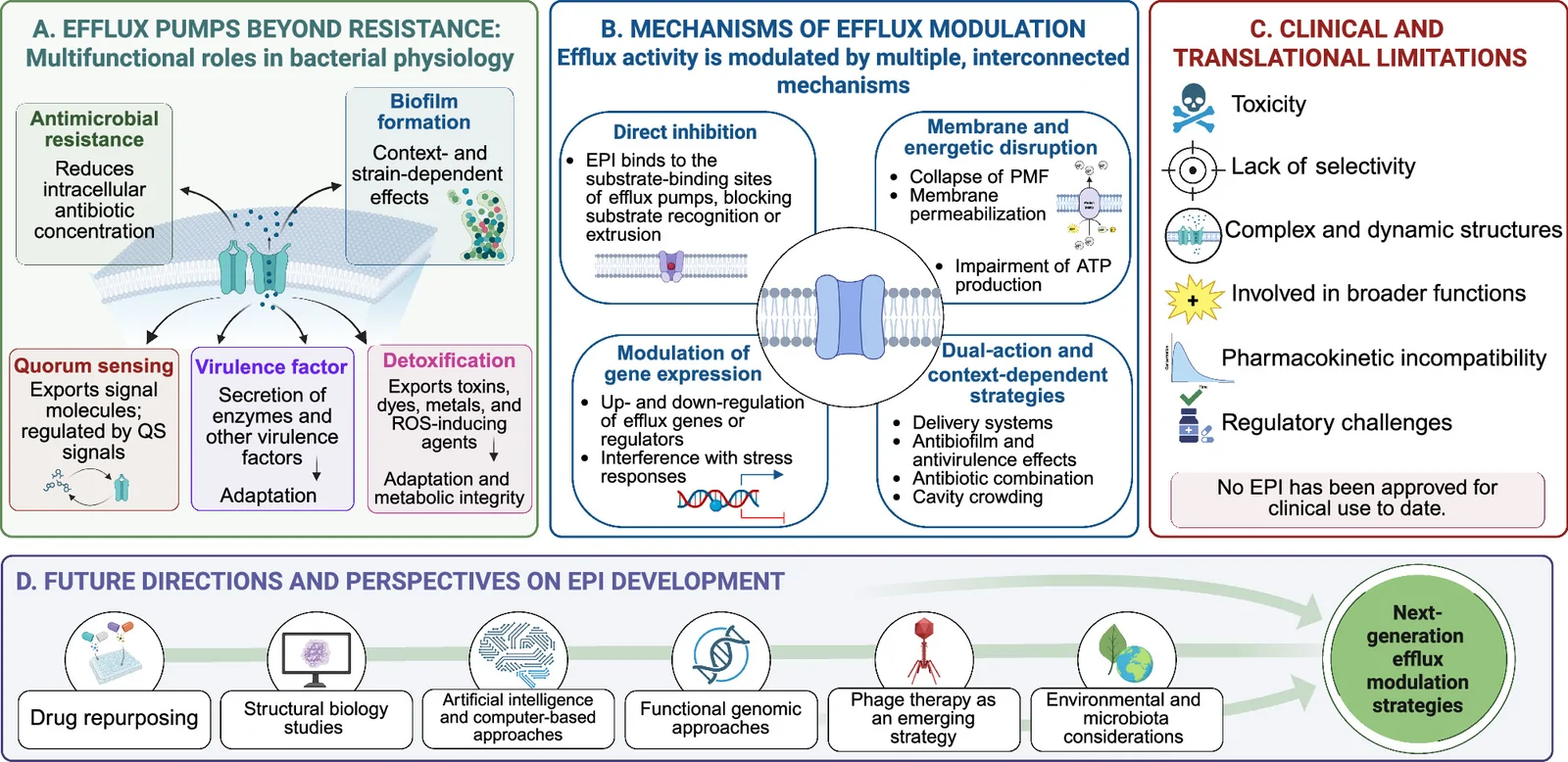
Efflux pumps: roles beyond antimicrobial resistance, mechanisms of efflux modulation, translational challenges, and future directions. Panel A illustrates the diverse physiological roles of efflux pumps, including biofilm formation, quorum sensing, virulence factor secretion, and detoxification. Panel B depicts the different mechanisms of action of efflux pump inhibitors (EPIs), including direct pump inhibition, membrane and energetic disruption, modulation of gene expression, and dual-action effects through interference with additional bacterial cellular processes. Panels C and D summarize the major limitations that have hindered the clinical translation of EPIs and the emerging strategies being explored to overcome these challenges. ATP, adenosine triphosphate; EPI, efflux pump inhibitor; PMF, proton motive force; QS, quorum sensing. Created with https://BioRender.com.

Beyond resistance, efflux pumps are increasingly recognized as multifunctional adaptive systems that play diverse roles in bacterial physiology and virulence (Fig. 1, panel A). These transporters have been implicated in processes like biofilm formation^8,9^, although their contribution appears to be highly context- and strain-dependent, with studies showing no correlation^10,11^ or a negative association^12^ with biofilm formation. Similarly, efflux pumps are functionally interconnected with quorum sensing (QS) as they can export signal molecules or their precursors^13–15^ and be regulated by QS molecules^16,17^. Importantly, the roles of efflux pumps in detoxification and virulence factor secretion, including QS molecules and phenazine, support bacterial adaptation and metabolic integrity under environmental- and host-imposed stress conditions^18–21^. This places efflux pumps as broad regulatory and physiological systems, challenging the view of these transporters as solely resistance determinants and complicating efforts to target them through direct inhibition.

Despite extensive efforts to target efflux pumps and the exploration of several classes of efflux pump inhibitors (EPIs)^1,22^, no EPI has been approved for clinical use. In this context, the increased toxicity and reduced specificity^23–25^ associated with the intrinsic properties of these transporters, as described above, and the lack of clinically relevant evidence that pump modulation produces sustained intracellular antibiotic exposure further complicate their translation into clinical use. As a result, efflux pump inhibition strategies should account for the dynamic nature and functional integration of efflux activity. In this Perspective, we explore efflux-targeting strategies beyond direct inhibition. Then, we examine the biological and pharmacological barriers that have limited EPI translation and propose a decision-oriented framework linking mechanistic validation, intracellular drug accumulation, exposure-response relationships, combination selection, dosing, scheduling, and resistance risk. The central question is therefore not simply whether a compound inhibits efflux, but whether it produces a clinically achievable and therapeutically meaningful improvement against a defined bacterial population.

### Mechanisms of efflux inhibition and modulation

Here, efflux modulation refers to the interventions that alter the contribution of bacterial efflux to antimicrobial susceptibility. Direct EPIs physically interfere with substrate recognition, whereas indirect modulators alter membrane energetics or integrity, regulate efflux gene expression, reduce antibiotic recognition as efflux substrates, or affect multiple bacterial mechanisms. These mechanisms differ substantially in their mode of action and are, therefore, discussed separately in this section (Fig. 1, panel B; Table 1).

**Table 1:** Representative examples of direct efflux inhibition, efflux modulation, and efflux-evasive strategies

Category	Representative compounds/strategies	Target organism(s)	Proposed efflux-related effect	Key limitation/observation	Ref.
Direct efflux pump inhibitors	Reserpine	*S. aureus*	Direct inhibition of NorA (MFS family), increasing intracellular accumulation of fluoroquinolones and ethidium bromide	Strong EPI activity but unsuitable clinically due to toxicity	^26–28^
	*p*-Coumaric acid derivatives	*P. aeruginosa*	Proposed inhibition of MexAB-OprM	Evidence largely based on docking and potentiation assays	^29^
	Pyranopyridine derivatives	*P. aeruginosa*	Direct inhibition of efflux transport; potentiation of aztreonam	Limited mechanistic validation; early-stage development	^30^
Energy uncouplers and membrane-active compounds	CCCP	ESKAPEE pathogens	Dissipation of proton motive force reduces energy-dependent efflux	Experimental control; high toxicity precludes clinical use	^31^
	PAβN	Primarily Gram-negative bacteria	Permeabilizes the outer membrane and reduces RND-mediated efflux	Also permeabilizes membranes, complicating interpretation	^32,33^
	Daptomycin	*S. aureus*	Membrane depolarization may indirectly affect energy-dependent transporters	Direct inhibition of specific pumps not established	^34–36^
Regulatory modulation of efflux expression	BING peptide	*P. aeruginosa*	Downregulates *mexB*, *mexY*, and *oprM* via CpxR	Changes in transcription may not reflect transporter activity	^37^
	Cec4 peptide	*A. baumannii*	Induces expression of multiple RND-associated genes	Stress caused to the envelope promotes gene regulation	^38^
	Polymyxin	*A. baumannii*	Induces expression of multiple RND-associated genes	Regulatory response may contribute to tolerance	^39^
	Esc(1–21)c	*P. aeruginosa*	Downregulates MexAB-OprM and upregulates OprD, promoting antibiotic accumulation	Combined effects on influx and efflux make attribution difficult	^40^
	Ellagic acid magnetic nanoparticles	*E. coli*	Reduced *acrB* and *tolC* expression	Effect may be nanoparticle- and context-specific	^41^
	Silver nanoparticles	*A. baumannii*	Downregulation of *ade* efflux genes and reduced efflux activity	Requires validation beyond transcriptional analyses	^42^
Dual-action and context-dependent strategies	Gentamicin or erythromycin liposomes + PAβN	*P. aeruginosa*	Improved intracellular delivery and reduced virulence traits	Multiple simultaneous mechanisms involved	^56^
	PAβN, NMP, thioridazine	*E. coli*, *K. pneumoniae*, *S. aureus*	Efflux inhibition associated with reduced biofilm formation	Contribution of efflux modulation versus other effects remains unclear	^44^
	Quercetin	Carbapenem-resistant Gram-negative bacteria	Simultaneous inhibition of carbapenemases and efflux activity	Multifactorial mechanism complicates interpretation	^43^
	Fluoroquinolone derivatives	Enterobacterales	Occupies overlapping binding trajectories within AcrB	Ligand co-occupancy depends on several factors	^45^

### Direct inhibitors of efflux transport

Direct inhibition refers to the ability of an EPI to bind to the substrate-binding sites of efflux pumps, blocking substrate recognition or extrusion independently of changes in membrane integrity or efflux gene regulation^1^. Among the best-characterized examples of direct efflux inhibition is the plant-derived alkaloid reserpine, which inhibits efflux pumps of the Major facilitator superfamily (MFS) family, most notably the NorA efflux pump in *S. aureus*, resulting in increased intracellular accumulation of fluoroquinolones and ethidium bromide^26^. Due to its strong and reproducible EPI effect, reserpine has been widely used as a positive control in efflux pump inhibition assays; however, it has not advanced into clinical application due to its toxicity^27,28^.

Although considerable effort has been made to discover new direct inhibitors, most candidates remain in the early stages of development. In many cases, efflux activity is inferred from docking analyses and antibiotic potentiation assays rather than from direct mechanistic validation. Representative examples include the plant-derived compound p-coumaric acid and its derivatives as potential inhibitors of *P. aeruginosa* MexAB-OprM^29^ and the optimization of pyranopyridine^30^ and pyridopyrimidine^31^ derivatives. Notably, a pyranopyridine derivative exhibited increased efflux pump inhibition in vitro and in vivo in a rat model of *P. aeruginosa* pneumonia when combined with aztreonam^32^, illustrating the translational potential of this strategy despite its current limitations.

However, a major player in the development of direct EPI is that some efflux pumps, such as RND systems, are large, dynamic, and highly promiscuous transporters with flexible binding sites, making selective and durable inhibition difficult^33^. As a result, direct inhibition is unlikely to represent a broad effective strategy, reinforcing the need to consider alternative approaches based on efflux modulation.

### Energy uncouplers and membrane-active compounds

Many efflux pumps rely on the proton-motive force (PMF) and the membrane potential to transport substrates. Therefore, the disruption of proton gradients, membrane potential (ΔΨ), or membrane integrity has been widely explored as a strategy against efflux activity. Carbonyl cyanide m-chlorophenylhydrazone (CCCP) is a well-characterized protonophore that dissipates the PMF across bacterial membranes. Several studies have shown that CCCP can increase antibiotic susceptibility or intracellular compound accumulation in ESKAPEE pathogens, including *A. baumannii*^34^, *K. pneumoniae*^35^, *P. aeruginosa*^36^, and *S. aureus*^37,38^. However, similar to reserpine, CCCP is primarily used as an experimental control^39^ since its elevated cytotoxicity has hampered its progression into clinical use^40,41^. Similarly, phenylalanine-arginine β-naphthylamide (PAβN) is also widely used as a reference compound in efflux assays. Nevertheless, PAβN can permeabilize the outer membrane in addition to interfering with RND-mediated efflux, making it difficult to attribute antibiotic potentiation exclusively to direct pump inhibition^42,43^.

Lipopeptides such as daptomycin dissipate the membrane potential and trigger cell wall stress in *S. aureus*^44–46^, which may influence multiple membrane-associated processes, including energy-dependent transporters such as MFS/ABC transporters. However, direct evidence that daptomycin enhances antibiotic accumulation by inhibiting specific efflux pumps remains limited. Therefore, membrane depolarization alone should not be considered evidence of efflux inhibition unless changes in pump activity or intracellular drug accumulation are directly demonstrated^44–46^. Overall, the contribution of energy uncoupling and membrane perturbation to antibiotic potentiation should be interpreted within the broader context of bacterial physiology and validated through direct measurements of efflux activity.

### Indirect modulation of efflux expression or regulation

Unlike direct EPIs, some antimicrobial agents and efflux-modulating compounds affect efflux activity at the transcriptional level by up- or downregulating genes and regulatory pathways, leading to context-dependent increases or decreases in efflux activity. Exposure to membrane-active or stress-inducing agents frequently leads to the upregulation of efflux systems as part of an adaptive response. For instance, polymyxin induced the expression of several RND-related genes, including *bpeB*, G4881, G4899, G1811, G4673, G1546, and G3649 in *A. baumannii*^47^. Similarly, the antimicrobial peptide Cec4 upregulated *macAB* and a gene encoding an RND efflux transporter permease subunit in *A. baumannii*^48^. In contrast, some antimicrobial agents downregulate efflux pump expression, as observed for the peptide BING, which downregulates *mexB*, *mexY*, and *oprM* in *P. aeruginosa* by modulating the envelope stress regulator CpxR, linking regulatory interference and reduced efflux activity^49^.

However, modulation of efflux gene expression does not always correlate with improved antimicrobial susceptibility. For example, screening of a natural product-like library identified coumarin-like, 1,4-benzodioxan-like, and 4-chloroindole compounds that act as both substrates and inducers of the MexAB-OprM and MexCD-OprJ efflux systems in *P. aeruginosa*. In addition to inducing efflux pump expression, the compounds also reduced virulence-associated phenotypes, including swarming motility, type III secretion system-mediated exotoxin production, and *Caenorhabditis elegans* killing, highlighting the complex interplay between efflux regulation and bacterial pathogenicity^50^. Similarly, overexpression of MexAB-OprM in *P. aeruginosa* reduced the production of QS autoinducers and QS-regulated virulence factors^51^, while the MexEF-OprN efflux pump was shown to export the PQS precursor HHQ, thereby modulating QS and virulence-associated phenotypes^52^. These findings illustrate how efflux systems can influence both antibiotic resistance and pathogenic behavior.

Efflux modulation can also alter the balance between influx and efflux; for example, the antimicrobial peptide Esc(1-21)c downregulates the efflux system MexAB-OprM while upregulating the OprD porin of *P. aeruginosa*, increasing intracellular antibiotic accumulation through the combined effects of uptake and extrusion^53^.

In general, regulatory responses are highly context-dependent, as the same compound may induce or repress efflux systems depending on the nature of the stress, compound, bacterial strain, growth conditions, and the characteristics of the EPI delivery system. For instance, ellagic acid-associated magnetic nanoparticles exhibited enhanced anti-*E. coli* activity and reduced the expression of *acrB* and *tolC*^54^. Similarly, biologically synthesized silver nanoparticles downregulated the efflux-associated genes *adeA, adeC, adeS, adeR, adeI, adeJ*, and *adeK* in multidrug-resistant *A. baumannii* and inhibited efflux activity in ethidium bromide accumulation assays, suggesting that efflux modulation may contribute to their antibacterial effects^55^. However, such effects should not be generalized across different nanoparticle compositions, pathogens, or experimental conditions. Moreover, changes in efflux gene expression do not necessarily translate into proportional changes in transporter activity or intracellular antibiotic accumulation. Therefore, transcriptional analyses should be complemented by functional efflux assays, membrane-integrity controls, and direct measurements of intracellular drug accumulation to establish whether regulatory modulation produces a therapeutically meaningful effect.

### Dual-action and context-dependent strategies

Efflux modulation is often accompanied by broader physiological effects that collectively enhance antimicrobial activity. Consequently, some EPIs and delivery systems can simultaneously influence antibiotic accumulation, resistance mechanisms, and virulence-associated traits, providing benefits that extend beyond efflux inhibition alone. For instance, liposomes loaded with gentamicin and erythromycin in combination with PAβN exhibited greater antimicrobial activity against *P. aeruginosa* than the corresponding free antibiotics, while simultaneously reducing virulence-associated phenotypes, including protease, lipase, pyoverdine, and pyocyanin production, as well as motility and biofilm formation. The proposed mechanism involves liposome fusion with and disruption of the bacterial membrane, facilitating intracellular delivery of both antibiotics and the efflux pump inhibitor^56^.

Similarly, efflux inhibition may affect bacterial traits beyond antimicrobial resistance. PAβN, 1-(1-naphthylmethyl)-piperazine (NMP), and thioridazine reduced biofilm formation in *E. coli*, *K. pneumoniae*, and *S. aureus*, supporting a role for efflux systems in biofilm development and highlighting the potential of EPIs to simultaneously impair resistance- and virulence-associated phenotypes^57^. Likewise, the flavonoid quercetin was shown to exhibit dual activity by inhibiting carbapenemases and efflux pump activity in carbapenem-resistant Gram-negative bacteria. Mechanistically, quercetin forms stable complexes with carbapenemases and occupies positions similar to those of the antibiotic meropenem, placing itself near the enzyme’s catalytic residues and engaging the same amino acids involved in meropenem binding^58^. On the one hand, these effects reinforce the use of EPIs as adjuvants and show how they may simultaneously target distinct resistance and virulence mechanisms, highlighting their potential value in antimicrobial therapy. On the other hand, because these effects often arise from multiple simultaneous mechanisms, it may be difficult to determine the relative contribution of efflux modulation to the overall antimicrobial outcome.

At a different level, structural competition within the transporter may influence substrate recognition and extrusion. High-resolution structural studies have shown that the large and flexible binding cavities of RND transporters, including AcrB and MexB, can accommodate multiple ligands and support overlapping steric, hydrophobic, and π-stacking interactions^59–61^. These observations have led to the hypothesis that simultaneous occupancy by different substrates, sometimes described as cavity crowding, may alter ligand residence time, conformational transitions, or transport kinetics. Consistent with this concept, bulky antibiotic scaffolds, including selected macrolide, rifamycin, and fluoroquinolone derivatives, can establish extensive interactions within RND transporter binding pockets and may be extruded less efficiently than smaller or more conformationally flexible compounds^62,63–66^. For example, fluoroquinolones bearing bulky C7 substituents in Enterobacterales have been proposed to occupy overlapping binding trajectories within AcrB, providing a potential model for how cavity occupancy may influence substrate handling independently of changes in efflux gene expression^45^. However, the consequences of ligand co-occupancy are likely to depend on transporter identity, substrate concentration, binding-site geometry, ligand affinity, and the sequence and timing of exposure.

Cavity crowding should therefore be regarded as a structure-based and experimentally testable hypothesis rather than as an established mechanism of therapeutic efflux modulation. Demonstrating its relevance would require direct measurements of transport kinetics, intracellular antibiotic accumulation, and combination activity under conditions in which membrane permeability, PMF, and efflux pump expression remain unchanged. If experimentally validated, this phenomenon could provide a framework for designing antibiotic combinations or molecular scaffolds that exploit transporter occupancy without functioning as conventional direct efflux pump inhibitors.

### Clinical and translational challenges and limitations

Despite decades of intensive research and substantial evidence of in vitro activity, together with more limited evidence from in vivo studies, efflux pump inhibitors have largely failed to progress from promising preclinical candidates to clinically available antimicrobial adjuvants^67–69^. This translational gap reflects a combination of biological, pharmacological, and regulatory challenges that undermine the clinical viability of these compounds (Fig. 1, panel C). In this context, one of the most important limitations is the lack of target selectivity, as exemplified by the reference EPIs CCCP and PAβN^1,70^, which, in addition to enhancing intracellular antibiotic accumulation, may also interfere with host transport systems, potentially altering drug pharmacokinetics, promoting drug-drug interactions, and increasing the risk of adverse effects^71–73^.

The biological organization of efflux systems further complicates therapeutic targeting. In this context, efflux pumps are embedded within broader stress-response networks, and their inhibition can trigger compensatory responses that promote bacterial adaptation^70^. Moreover, although EPIs are designed to suppress resistance and increase bacterial susceptibility, studies have shown that bacteria exposed to EPI or EPI-antibiotic combinations can still select for resistance-associated mutations or resistance to the inhibitor^74,75^. These outcomes demonstrate that short-term reductions in minimal inhibitory concentration (MIC) or dye extrusion are insufficient to establish durable therapeutic benefit and that efflux-targeting strategies must also be evaluated under conditions that capture bacterial adaptation and resistance evolution.

Pharmacokinetic limitations constitute another important barrier to clinical translation. Many EPI candidates exhibit poor bioavailability, chemical instability, extensive protein binding, rapid metabolic clearance, or inadequate penetration into infected tissues, preventing them from reaching and maintaining effective concentrations at the site of infection^22,76,77^. Unlike conventional antibiotics, efflux modulators generally depend on combination with an active antimicrobial drug and therefore require compatible tissue distribution and sufficiently overlapping exposure profiles^74,78^. Achieving this level of coordination has proven difficult and has driven interest in advanced formulation strategies, including liposomal encapsulation, polymeric nanoparticles, and targeted delivery systems designed to improve local concentration while minimizing systemic exposure^79,80^.

From a regulatory perspective, efflux modulators present additional challenges because they are generally developed as antibiotic adjuvants rather than as standalone antimicrobial agents. Consequently, their clinical development must demonstrate therapeutic benefit in combination with a specific antibiotic partner, while also considering the pharmacokinetic and pharmacodynamic relationships between both components^78,81^.

To address these challenges, the development of efflux-modulating strategies should follow a structured translational framework that integrates mechanistic validation, pharmacokinetic and pharmacodynamic considerations, resistance-risk assessment, and safety evaluation (Table 2). Key questions include whether efflux is clinically relevant in the target pathogen, whether the selected antibiotic is an efflux substrate, whether potentiation results from specific modulation of efflux activity rather than nonspecific membrane effects, and whether clinically achievable exposures of both components can be synchronized at the infection site. Additional considerations include optimization of administration schedules, evaluation of resistance suppression, and assessment of toxicity associated with host-transporter interactions. Together, these criteria provide a basis for explicit go/no-go decisions and help determine whether development should proceed through direct efflux inhibition, alternative delivery platforms, or efflux-evasive antimicrobial strategies^22,82–86^.

**Table 2:** Decision-oriented framework for the preclinical and translational development of efflux-modulating therapies

Development question	Evidence required	Recommended approach	Decision supported	Ref.
Is efflux clinically relevant in the target pathogen?	Pump expression, genetic validation, functional efflux and intracellular accumulation data	Knockout or knockdown studies, efflux assays, clinical-isolate profiling	Target pathogen and patient population	^76,84^
Is the antibiotic a relevant efflux substrate?	Pump-dependent changes in susceptibility and intracellular exposure	Accumulation assays, isogenic strains, transport studies	Antibiotic-partner selection	^87,88^
Is potentiation mechanistically specific?	Separation of efflux modulation from membrane damage, increased influx, or independent antibacterial activity	Membrane-integrity controls, concentration–effect analysis, direct transport measurements	Mechanistic validation	^82,86^
Are effective concentrations clinically achievable?	Unbound concentrations of both components in plasma and infected tissues	Population pharmacokinetics and infection-site exposure modelling	Dose selection and route of administration	^22,76^
Are the exposures of both agents synchronized?	Overlapping concentration–time profiles at the infection site	Joint pharmacokinetics and pharmacodynamics modelling and dynamic infection models	Dose ratio and formulation strategy	^76,84^
Which administration sequence is optimal?	Onset, duration and reversibility of efflux modulation	Simultaneous, sequential and intermittent exposure studies	Treatment schedule	^86,88^
Does the combination suppress bacterial regrowth and resistance?	Kill kinetics, resistant subpopulations and serial-exposure outcomes	Time-kill, hollow-fibre or dynamic PK infection models	Resistance-risk assessment	^84,86^
Is the therapeutic window acceptable?	Mammalian transporter interactions, mitochondrial effects and systemic toxicity	Host-transporter panels, mitochondrial assays and in vivo safety studies	Go/no-go decision	^85^
Is direct inhibition the best strategy?	Comparison with efflux-evasive chemistry or targeted delivery	Comparative translational assessment	Continue, redirect or terminate development	^82,83^

### Future directions and perspectives on EPI development

Drug repurposing has emerged as an attractive approach to overcome the challenges associated with drug development timelines, including those targeting efflux systems. In this context, clinically approved compounds with established safety and pharmacokinetic profiles that exhibit previously unrecognized efflux-modulating activity have been explored, including non-steroidal anti-inflammatory drugs^89,90^, anti-cancer^91^, and antidepressants^92,93^. While repurposing does not circumvent all limitations, it offers an alternative route to de-risk early development and accelerate clinical evaluation.

Structural studies have advanced our understanding of efflux systems in Gram-negative bacteria, including the *E. coli* AcrAB-TolC^94,95^. Crystallographic analyses, including the structures of AcrB and MexB in complex with inhibitors reported by Nakashima and colleagues, provided key insights into inhibitor binding and pump interference, helping to clarify previously unresolved aspects of EPI modes of action^96^. Furthermore, cryo-electron microscopy and crystallography have shown that efflux pumps change shape during substrate recognition, binding, and transport. These studies also reveal flexible and adaptable binding regions capable of interacting with different types of molecules^62,97,98^. However, despite the alternative inhibition strategy offered by these methodologies, given the limitations of EPIs’ clinical application, chemical optimization alone is unlikely to be sufficient, and EPI development requires an integrated approach^99^.

Artificial intelligence is currently enabling structure-guided discovery at a scale that is difficult to achieve through traditional screening approaches^100^. Deep learning models trained on structural, physicochemical, and biological datasets can rapidly identify novel scaffolds compatible with efflux pump binding sites and predict ADMET liabilities early in development^100,101^. In parallel, generative approaches are being used to design de novo molecules tailored to specific targets, including transient binding sites identified by cryo-EM or molecular dynamics studies^102,103^.

Emerging biological approaches, particularly bacteriophage-based strategies, further expand the EPI landscape. Engineered phages can be designed to selectively target efflux-overexpressing bacteria or deliver gene-editing tools that disrupt efflux gene expression^104,105^. In preclinical infection models, phage-antibiotic combinations have demonstrated the ability to reverse resistance phenotypes and significantly reduce bacterial burden^106,107^. Although regulatory frameworks for phage therapeutics remain under development, their integration with EPI-based strategies represents a future direction.

At the systems level, functional genomics approaches are increasingly important for identifying vulnerabilities linked to efflux regulation. Transcriptomic profiling, CRISPR interference screens, and synthetic lethality analyses have revealed complex regulatory networks connecting efflux pumps to metabolic pathways and stress responses^108,109^. Targeting genes or gene expression regulators provides robust alternatives to direct pump inhibition. Indeed, high-throughput mutant library screenings have proved important tools for the identification of genes and pathways involved in antimicrobial resistance, providing insights into agents’ modes of action and resistance mechanisms^110,111^.

Additionally, environmental and microbiota considerations must be incorporated early in EPI development. Compounds with high environmental persistence may exert unintended selective pressures in wastewater or soil ecosystems, potentially promoting efflux-mediated resistance outside clinical settings^112^. Similarly, broad-spectrum EPIs may pose a risk of disrupting commensal microbiota. While this effect has not been fully characterized for most efflux inhibitors, extensive evidence from antibiotic therapy indicates that broad antimicrobial exposure can significantly alter microbial community structure and diversity^78,113^. The integration of green chemistry principles and microbiome-dependent preclinical models will be essential for clinical translation.

Beyond developing classical EPIs, an alternative strategy is to design antibiotics that are intrinsically less susceptible to efflux recognition. For instance, the anionic character of delafloxacin and its pH-dependent ionization contribute to enhanced antibacterial activity under acidic conditions and may reduce its susceptibility to extrusion compared with other fluoroquinolones. However, resistance can still emerge through efflux pump overexpression and additional target-associated mechanisms^114–118^.

In summary, the future of EPI development depends less on identifying single breakthrough molecules and more on integrating structural biology, computational design, functional genomics, and considerations of host and environmental effects (Fig. 1, panel D). By combining these approaches, EPIs may finally transition from repeated preclinical failures to viable therapeutic adjuvants that extend the lifespan of existing antibiotics.

## Conclusions

Efflux pumps are not isolated resistance determinants but components of broader physiological and regulatory networks that influence antimicrobial exposure, stress adaptation, virulence, and biofilm-associated phenotypes. To date, EPIs have largely been constrained by systemic toxicity, low target selectivity, and poor translational progression. Still, the current landscape shows that efflux modulation operates through several mechanisms, including direct inhibition, envelope and bioenergetic perturbation, transcriptional reprogramming, and multi-mechanism interactions. Therefore, the therapeutic value of efflux modulation will ultimately depend on whether it produces a sustained increase in antibiotic exposure at clinically achievable concentrations against a defined pathogen-pump-drug context. Integrated evaluation of intracellular accumulation, pharmacokinetic compatibility, dose ratio, administration sequence, host-transporter interactions, and resistance emergence should therefore guide development decisions. Such a framework may help distinguish combinations with realistic clinical potential from those that demonstrate only nonspecific in vitro potentiation and may also identify situations in which antibiotic redesign or targeted delivery is preferable to direct pump inhibition.

## Data Availability

No datasets were generated or analysed during the current study.
